# Eye, Ear, Nose and Throat

**Published:** 1900-12

**Authors:** 


EYE, EAR, NOSE AND THROAT. A Manual for Students and Practitioners. By Wm. L. Ballinger, M.D., Assistant Professor of Otology, Rhinology and Laryngology in the College of Medicine of the University of Illinois; College of Physicians and Surgeons, Chicago; and A. G. Wippern, M.D., Professor of Ophthalmology and Otology, Chicago Eye, Ear, Nose and Throat College. Published by Lea Brothers & Co., Philadelphia and New York.

This manual on diseases of the eye, ear, nose and throat will prove a very handy work for medical students, but cannot be of great value to practitioners of medicine. Students frequently need just such a work, embracing as it does all the four organs, because it is much more handy and much less expensive than to have a separate treatise on each department. The authors have done their work well, and the book contains numerous and well adapted illustrations. The anatomical plates from the well-known work of Testut are of course marvels of beauty. The work is more like a quiz-compend than a manual with the questions left out. The only trouble as we see it, is the fact that if a student or practitioner is compelled to have a book on the eye, ear, nose and throat, he wants to spend the money for one that is more complete and which will be an addition to his accumulating library. We think that students, and as for that matter, practitioners also, ought to spend money for books with the idea that such are purchased, not for the day, but for some time to come, and as such should be the best and most complete. No doubt this manual will meet the demands of many and, as such, will find a ready sale. The authors have given a text safe to follow. It is a work thoroughly up-to-date and thoroughly well written. The publishers, as they always do, have executed their part in a most satisfactory manner. ROY.

